# Expulsion of a Mass of Hair from the Uterus

**Published:** 1842-09

**Authors:** Henry R. Frost

**Affiliations:** Charleston, South Carolina


*Expulsion of a mass of hair from the Uterus.*—By Henry R. Frost, M. D., Charleston, South Carolina.—To give full interest to the above extraordinary occurrence, it is necessary to detail the circumstances connected with a case of tedious and instrumental labor.

A colored girl aged 25 years, pregnant with her first child, was taken with the pains of labor, about 6 o'clock on the evening of the 9th of September, 1838. The habit of the patient was strong, and her health good. There was no occurrence during the first twenty-four hours to excite uneasiness, but the delay which occasioned the accoucheuse to suppose that there was something wrong.

On the evening of the 10th, I was called to see her. Upon examination, I discovered nothing at fault, but the slowness of dilatation in the os uteri. There appeared to be some thickening of its orifice. The liquor amnii was passing off slowly, the hairy scalp could be felt protruding, and the vertex resting upon the brim of the pelvis. To favor relaxation, a dose of castor oil had been administered in the morning, and a small quantity of blood taken from the arm. Expecting that the pains would be renewed, and finding nothing requiring my assistance, left the patient in charge of the midwife.

*Sept.* 11*th*.—This morning at 9 o'clock, find upon examination, that the vertex had made little progress; the os uteri being a little dilated, and thinner; repeated the venesection.

1 o'clock.—But little progress made, and as the pains were exhausting without producing much effect, recommended that they be allayed as much as possible; directed acetate of morphia in doses of one-sixth of a grain every half hour until easier.

7 o'clock.—The medicine had been taken, and some relief experienced.

12*th*.—The pains were removed during the night, but with little benefit. Upon examination, but little change was perceptible; the head had descended a little lower, and the protruding scalp filled up the os uteri. Apprehending that there might be some obstruction in the passage of the head, and that difficulty would occur in the course of the labor, requested assistance.

The late Professor Wagner was called, and upon examination of the patient, concurred in the opinion I had formed of the presentation; recommended patience and perseverance. The powers of the patient were good, and as the pulse was more active than proper, further venesection was recommended.

The condition of the patient was little altered during the day and night.

13*th.*—Still trusting to nature, and nothing done to expedite delivery. At 8 o'clock P. M., the forceps were applied, but with no advantage. In the course of the night, the ergot in infusion was freely administered, but without any effect.

During the day a discharge of meconium in considerable quantity was noticed, an occurrence in a presentation of the vertex very unusual.

In the progress of the labor, it became necessary to draw off the urine twice a day, but it is somewhat singular, that though the catheter was introduced, a very small quantity of urine was removed, while a tumor which was formed by the distended bladder could not be reduced, though pressure was made upon it.

14*th.*—At 9 o'clock, A. M. the head was perforated and the child extracted. The operation was completed in about half an hour, and was well supported by the patient. The placenta was extracted, and the patient made as comfortable as possible; some soreness and tumefaction was experienced, and at night nearly two quarts of urine were removed.

15*th*, 16*th*, 17*th.*—The patient continues to improve.

*Nov.* 17*th.*—The progress of the patient was a very slow, but gradual amendment. About this period, she complained of fever, which continued at variable periods during the day, followed by very profuse perspiration. There was considerable discharge from the vagina of a puriform and very offensive fluid, pains about the region of the pubes at times considerable, occasionally tumefaction of the abdomen, the appetite variable. To relieve some of these symptoms, general treatment was adopted, and the use of an astringent injection.

19*th.*—The patient informed me that the vagina was filled with a substance, which prevented the introduction of the syringe, that sitting was very uncomfortable, and the feeling of something which was to be removed.

Upon examination, I discovered a substance of a dark color, presenting at the os externum. I provided myself with a pair of forceps, and with little trouble extracted a mass, several inches in length (say 5 inches), and an inch and a half in diameter, at its largest part, looking like wet tow, of an irregular and somewhat pear-like shape. Carefully inspecting it, it proved to be a mass of hair, in short pieces, of an inch to two inches in length, very offensive, and saturated with a purulent looking fluid. The symptoms above noticed soon subsided, and the person recovered in a considerable degree. The preparation is now to be seen in the museum of the Medical College of the State of South Carolina.

The preceding occurrence affords a subject for speculation and inquiry.

It could not have been an offcast from the first child, the quantity of hair being too considerable, and of a different texture from what is usually found on a colored child.

It must have existed in the uterus at the time the first child was born, and must have remained in the cavity of this organ, and the vagina, nearly two months after the delivery. This is more remarkable, inasmuch as the placenta was removed after the birth of the child, and the condition of the uterus carefully examined.

When removed, it had no appearance of being enveloped with membranes.

Was it an abortive effort of nature in the production of twins? or was it of that class of morbid growths, which are so frequently formed in the ovaria?—*Am. Jour. Med. Sci.*

